# The Spectrum of Familial Hypercholesterolemia (FH) in Saudi Arabia: Prime Time for Patient FH Registry

**DOI:** 10.2174/1874192401711010066

**Published:** 2017-07-26

**Authors:** Faisal Alallaf, Fatima Amanullah H.Nazar, Majed Alnefaie, Adel Almaymuni, Omran Mohammed Rashidi, Khalid Alhabib, Fahad Alnouri, Mohamed-Nabil Alama, Mohammad Athar, Zuhier Awan

**Affiliations:** 1 Department of Medical Genetics, Faculty of Medicine, Umm Al-Qura University, Mekkah. Saudi Arabia; 2 Department of Biology, Genomic and Biotechnology Section, Faculty of Science, King Abdulaziz University, Jeddah. Saudi Arabia; 3 Department of Clinical Biochemistry, Faculty of Medicine, King Abdulaziz University, Jeddah. Saudi Arabia; 4 Interventional Cardiology, King Fahad Cardiac Center, College of Medicine, King Saud University, Riyadh, Saudi Arabia; 5 Cardiovascular Prevention and Rehabilitation Unit, Prince Sultan Cardiac Centre, Riyadh, Saudi Arabia; 6 Adult interventional cardiology, Cardiology unit, King Abdulaziz University Hospital (KAUH), Jeddah, Saudi Arabia; 7 Department of Science and Technology, Umm Al-Qura University, Mekkah, Saudi Arabia

**Keywords:** Familial hypercholesterolemia, Cardiovascular disease, FH registry, Cascade screening, Mutations, Genetic screening

## Abstract

**Background:**

Familial hypercholesterolemia (FH) is a life-threatening inherited condition. Untreated patients have the risk to develop raised plasma levels of cholesterol, atherosclerosis and cardiovascular disease (CVD). If diagnosed and treated early in life, the pathological consequences due to atherosclerosis could be avoided and patients with FH can have an anticipated normal life. Mounting evidence suggests that FH is underdiagnosed and undertreated in all populations. The underlying molecular basis of FH is the presence of mutations in one or more genes in the low-density lipoprotein receptor (*LDLR*), apolipoprotein B (*APOB*) or proprotein convertase subtilisin/kexin 9 (*PCSK9*). However, their prevalence is largely unknown in Saudi Arabia but given the high rates of consanguinity, the prevalence appears to be higher. Furthermore, the high prevalence of obesity and diabetes mellitus in Saudi Arabia increases the vascular disease burden in FH cases by adding additional CVD risk factors.

**Objective:**

This article explores the spectrum of FH-causing mutations in the highly consanguineous Saudi community, the need for establishing the Saudi FH registry, the challenges in creating gene databases, and cascade screening.

**Conclusion:**

The establishment of FH registry and genetic testing should raise awareness not only among healthcare professionals, but the general population as well. It also helps to provide the best treatment regimen in a cost effective manner to this under-recognised population of FH patients.

## 1 FAMILIAL HYPERCHOLESTEROLEMIA (FH)

FH is a monogenic disorder of lipid metabolism that plays a key role in developing cardiovascular disease (CVD). Affected individuals have isolated high serum cholesterol and low-density lipoprotein-cholesterol (LDL-C) levels (1). The disease is primarily inherited in an autosomal dominant (AD) manner, but it also has an autosomal recessive AR mode of inheritance. Genetic mutations in one or more genes could be the cause of FH. The established AD genes are: low-density lipoprotein receptor (*LDLR*) [1], apolipoprotein B-100 (*APOB*) [2] or proprotein convertase subtilisin/kexin type 9 (*PCSK9*) [3]. Mutations in one allele of either of these genes may lead to the heterozygous FH (HeFH) phenotype and mutations in both alleles may lead to the more severe homozygous FH (HoFH) phenotype. The LDL receptor adaptor protein 1 (*LDLRAP1*) gene is responsible for the AR form of FH [4]. The AR form of FH has clinical similarity to both HeFH and HoFH with intermediate LDL-C levels if both alleles are affected. However, a normal lipid profile is seen in parents, hence the AR pattern, and a greater response to lipid-lowering treatment are achieved in these patients [5].

Considering that molecular defects occur in two alleles of the gene locus, HoFH individuals normally show a conspicuously increased amount of total serum cholesterol (>500 mg/dL, 13 mmol/L) and LDL-C levels (>450 mg/dL, 11.7 mmol/L) [6]. The accumulation of cholesterol leads to characteristic cutaneous lesions in the form of xanthoma tendinosum of the hands and feet, xanthelasmata around the eyelid and corneal arcus earlier in life [7, 8]. Myocardial infarction (MI) and sudden cardiac events can occur prematurely in adolescence due to atheroma of the coronaries, aortic root and valve [9]. Patients with a null mutation of the *LDLR* gene, resulting in no protein product, have a higher susceptibility to CVD than those mutations that alter the gene sequence and partially disrupts LDL receptor function [8, 10]. In contrast, HeFH commonly presents with lower serum cholesterol (250-450 mg/dL or 6.5-11.6 mmol/L) and LDL-C (200-400 mg/dL or 5.2-10.4 mmol/L) levels than HoFH [5]. They demonstrate the above clinical symptoms with slower progression of their disease. Patients with HeFH usually develop premature CVD before age 55 for men, and 60 for women. HeFH men tend to suffer from CVD than HeFH women probably due to sex-related hormonal factors [11].

## 2 CLINICAL AND LABORATORY DIAGNOSIS

Biochemical screening tests alone are not sufficient to confirm the presence of FH; this is due to the fact that cholesterol levels in the blood vary with several contributing factors such as, gender, age, ethnicity, certain drugs, physiological and pathological conditions. Indeed, the range of cholesterol values in FH cases overlap with non-genetic multifactorial hypercholesterolemia and may lead to a false negative or false positive diagnosis [12]. There are at least 4 widely used diagnostic systems used for the diagnosis of FH; the Simon Broome Register Group (United Kingdom) [13], the Dutch Lipid Clinic Network (DLCN) [14], the Make Early Diagnosis to Prevent Early Deaths (MEDPED) criteria (USA) and the one released by the European Atherosclerosis Society [15]. These diagnostic criteria rely mainly on; very high total cholesterol or LDL-C levels on repeated measurements. The Simon Broome and DLCN criteria, in addition to lipid parameters, take in consideration the presence of xanthomas, molecular diagnosis and a family history of some signs and symptoms of dyslipidaemia. Molecular genetic testing is based on direct DNA sequence analyses using a capillary method or high-throughput next-generation sequencing (NGS)-based method. Large insertions/deletions detection is usually done by the multiplex ligation-dependent probe amplification. Once a causative mutation is discovered in the index case, a cascade screening should be performed at least to the first degree relatives to demonstrate the dominant pattern of inheritance.

## 3 GENETICS OF FH

As mentioned earlier, mutations in one allele described above may lead to the HeFH phenotype while mutations in both alleles may lead to the HoFH phenotype. Few rare cases are ‘double heterozygotes’, *i.e.* they carry 2 different mutations of the aforementioned genes, which may lead to an intermediate phenotype between HeFH and HoFH [16]. Compound heterozygous mutations cases can also occur due to different allelic variations on the same gene loci.

To further illustrate the complexity of FH genotype, only loss-of-function mutations in the *LDLR* and *APOB* genes and only gain-of-function mutations in the *PCSK9* gene cause the disease. About 85% of FH cases are attributed to loss-of-function mutations in the *LDLR* gene [17]. More than 1700 different mutations in the *LDLR* (p13.1-p13.3) gene have been described so far [18] and registered in online gene databases http://www.ucl.ac.uk/ldlr/Current [19]. The *LDLR* database includes different types of mutations; single-nucleotide polymorphism, insertions/deletions, copy number variations and splicing sites defects. These mutations have the ability to disrupt the functional domains of the LDLR protein affecting its function to establish a normal initiative complex combining the receptor with the LDL-C particle.

Apolipoprotein B-100 (ApoB-100) is the ligand protein for the LDLR, encoded by the gene *APOB* that is mapped to the short arm of chromosome 2 (p24). *APOB* mutations account for <5% of FH cases [20]. Defects in this protein result in ligand affinity reduction for the LDLR, which results in poor clearance of LDL-C from the circulation allowing for hypercholesterolemia independent of the LDLR activity [21].

In less than a decade, mutations in a third gene located on the short arm of chromosome 1 (p34.1-p32) have been identified to be responsible for nearly 1% of monogenic FH cases [22]. The *PCSK9* gene produces an enzyme used to be known as “neural apoptosis-regulated convertase 1”, which has been associated with the LDLR protein degradation in the lysosome thus preventing it from recycling [3]. More than a handful of different gain-of-function mutations have been reported in the *PCSK9* gene which can cause increased degradation of LDLRs, minimising the numbers of receptors on the cell surface and causing monogenic FH.

*LDLRAP1* loss-of-function mutations result in a rare form of AR FH. The gene is mapped on the short arm of chromosome 1p35-36.1 [23]. In addition, genome-wide association studies have shown a strong association between Apolipoprotein E (*APOE*) p.Leu167del and the AD hypercholesterolaemia phenotype in a large family. Awan *et al.* [24] reported a mutation in the *APOE* gene associated with the classical autosomal dominant hypercholesterolemia (ADH) phenotype from a proband of an Italian origin which confirmed the observation of Marduel *et al.* and suggesting *APOE* gene as the 4th locus in ADH [25]. Other candidate genes have also been attributed to causing FH phenotype including *ABCA1, APOA2, APOC3, PON2, APOC2 and LPL*.

## 4 EPIDEMIOLOGICAL GENETICS OF FH

In most investigated Western populations, HeFH is perceived to affect approximately 1 in every 200 to 500 individuals [26] with an estimated one case in each million individuals for HoFH [26, 27]. However, recent studies have changed this perception and shown that FH is under diagnosed in most populations [14]. The Copenhagen General Population Study, a sample from a random European general population, included 69,016 individuals with HeFH diagnosed using the DLCN criteria. This finding suggested that HeFH prevalence according to DLCN criteria may reach 1 in every 200 individuals [14], or, for molecularly defined HeFH, one in 244 [28]. Consequently, HoFH may rise to one in every 300,000 individuals.

FH prevalence is greater with founder effects in geographically isolated regions, where the practice of consanguineous marriage increases the risk. Founder effects for FH were seen among French Canadians [29], Jews [30], South African Afrikaners [31], Lebanese [32], Indians [33], Tunisians [34], Icelanders [35] and Finns [36] among whom the frequency of *LDLR* mutations is higher than the general population. The Lebanese p.Cys681X mutation accounts for 82% of the FH cases in the Lebanese population [37]. Remarkably, the frequency of HoFH cases is 10-fold higher in Lebanon than in neighbouring nations.

Up to date, there is no genetic epidemiological study conducted for the frequency of FH in Saudi Arabia. Based on the 2015 population census (http://www.stats.gov.sa/en/node, Accessed April 2017), the predicted prevalence of FH in Saudi Arabia is expected to range from 158,712 to 63,485 cases of HeFH, based on the rate of 1:200-500, and 53 to 106 cases of HoFH based on the rate of 1:300,000-600,000 Fig. (**1**). The data available for the prevalence of FH in Saudi Arabia is limited due to the lack of national registries and genetic screening for FH. Currently, >80 clinically and molecularly confirmed HoFH are being treated with LDL-apheresis every 2 weeks in a single centre in Riyadh (capital of Saudi Arabia) and another centre is being initiated to deal with the increasing demand for such service (personal communication), which supports the predicted numbers given Fig. (**1**).

## 5 SPECTRUM OF MUTATIONS CAUSING FH IN SAUDI ARABIA

Only a few reports on the molecular aspects of FH were related to Saudi individuals (38)(39)(40)(41)(42). A systematic review to collect all FH-related mutations reported in the Saudi populations resulted in 21 variants mapped to 3 genes; *LDLR*, *APOB* and *PCSK9*. As expected, the majority of these variants were in the *LDLR* gene (>80%) Fig. (**2**), and only 2 mutations among these were found to be *novel* variants in the *LDLR* gene; c.1332dup (p.D445*) (39) c.2026delG (p.G676Afs*33) (41) .

On the basis of several studies, one should expect to find *APOB* and *PCSK9* variant in a smaller percentage of FH Saudi patients. Variation in the PCSK9 gene was found to be the second most common and variation in *APOB* gene were among the least common (Unpublished data; Dr Faisal Alallaf, Umm Al-Qura University: Medical genetic laboratories). No evidence of large deletions or duplications in the *LDLR* gene were associated with the presence of FH disease nor FH-related *LDLRAP1 or APOE* mutations were found.

From our clinical and molecular observation of FH cases, founder mutations were expected to originate from certain tribes in the central, northern and western region of Saudi Arabia [42].

The expected high prevalence of FH predicted from the high number of treated HoFH in Saudi Arabia, emphasise the urgent need for an FH registry as a preventive measure toward decreasing the number of HoFH. Indeed, both consanguineous and non-consanguineous high-risk individuals should be advised to have counselling and a genetic testing before having offspring and an immediate screening should be done for those children whom parents are clinically and molecularly confirmed FH cases.

## 6 MANAGEMENT OF FH

In contrast with secondary hypercholesterolemia, primary FH does not usually respond to diet. However, diet low in saturated fat is an important part of the management of FH together with lifestyle modification and smoking cessation. A common pharmacological strategy would be to combine statin-based treatment with other lipid-lowering medications with a different mechanism. For example, a statin might be combined with a cholesterol absorption inhibitor or a bile acid sequestrant which both work in the intestine to prevent cholesterol absorption [44, 45]. The FDA also approved PCSK9 inhibitor antibodies, neutralising circulating PCSK9, a protein associated with LDLR degradation in the liver for the treatment of FH patients [46]. These monoclonal antibodies are not expected to work in HoFH with *LDLR* null-mutations, given the mode of action of PCSK9 on the LDLR protein which requires a functional LDLR. LDL apheresis (or lipoprotein apheresis) can be used in all mutations causing HoFH as well as severe HeFH and pregnant FH patients. This bimonthly or monthly dialysis-like procedure clears the blood from excess lipoprotein. Apheresis treatment duration lasts between 2 to 3h and usually reduces the LDL-C level by about 70% [47]. Unfortunately, LDL particles increase over time, therefore, repeated treatment is required. At the present time, there is only 1 active LDL-apheresis centre in Saudi Arabia.

Recently, microsomal triglyceride transfer protein (MTP) inhibitors (lomitapide) and oligonucleotide developed against Apo-B 100 mRNA (mipomersen), have been approved in some countries for HoFH treatment [48]. However, the effectiveness of aforementioned drugs were not assessed in non-HoFH patients, and their effects on cardiovascular morbidity and mortality remain unknown. In addition, the cost effectiveness and safety of these treatments are inferior to LDL-apheresis and PCSK9 inhibitors, which may limit their future use.

Finally, liver transplantation is an effective, yet invasive, treatment for HoFH based on a small number of interventions [49]. This option works by replacing dysfunctional LDL receptors with functional ones, leading to nearly normal LDL metabolism [49]. Khalifeh *et al* [50] performed a successful liver transplantation to a HoFH patient, who was born to first-degree Lebanese cousins. Two of the most common complications following liver transplant are rejection and infection. Therefore, other treatments as stated above are now available which are more effective and less invasive.

## 7 THE NEED TO ESTABLISH A SAUDI FH REGISTRY 

The World Health Organization (WHO) defined patient registry as a collection of documents containing intrinsic/specific information about the state of health of individuals arranged in a systematic and comprehensive way, in order to serve a predetermined scientific, clinical or policy purpose [51]. The relatively low reporting of FH with the higher prevalence of CVD in Saudi Arabia [52] prompted us to initiate a patient database as a preventative measure toward reducing the incidence of CVD.

The FH registry aims toward raising awareness of FH among the population with the hope of decreasing the consequences of this condition in the long term and eventually lowering morbidity and mortality. Determining the exact cause and pathogenicity of the condition aids in preventing the complications. Moreover, understanding the genetic predisposition of such disorder provides an early intervention, thus early management. Furthermore, it will help determine the most common known mutation\variant among the Saudi population and identify population-specific novel mutations aiding future rapid molecular diagnosis. The database will allow different users to perform entries, including clinical and genetic service providers, to have a centralised system for ease of access from any location. Distinct privileges will be assigned to control access, yet the system will allow specific tasks related to education to be executed by general users. Researchers and other third parties, such as other registries, can access de-identified data through the consultative body, research ethics committees and the data stewards.

The careful documentation of FH cases will allow for statically-improved studies, scientific collaborations, meta-analytical studies, and international clinical trials to take place in Saudi Arabia. The outcome from this registry should enable studies of the natural history of the disease within Saudi individuals, provide evidence for therapies and suggest methods for health economic refinements.

## 8 BUILDING THE SAUDI GENETIC DATABASE

There is no standard basis for establishing FH registries since it is relatively an emerging field and each culture is unique. The International FH foundation lately set-up a very inclusive, well-designed, consensual guidance for FH managing based on an international viewpoint [53]. This can be a convenient guidance for the promotion of a patient database at all stages as a first step towards initiating a full registry that can benefit more parties.

Targeted, universal, and opportunistic screening strategies using the DLCN [54] or Simon-Broome [13] criteria will be used to identify index FH cases in the Saudi population. Both can be an appropriate standard for initial clinical FH detection and can be compared and evaluated in a 5-year period for efficiency within the Saudi population. In this way, suspected cases of FH could be referred to lipid specialists if available locally. Genotype and phenotype assessment are ideal for all FH cases, but the phenotype test should be sufficient even if genotype testing are not feasible. Data collection will be performed at various sites in the country, mainly from lipid clinics and laboratories where genetic results are reported. Creating a web-based platforms will enable the Saudi FH patient database to be very interactive and accessible from different locations, with multiple-level private access for a broad range of users. In order to cover more FH cases from the various region, additional pages can be added to the registry with user groups being assigned appropriate upload and access privileges. Thus, many general practices regardless of their location can also contribute to the FH patient database. General practitioner (GP) entries will be of special interest since each GP will examine a spectrum of FH cases and by virtue of such service, the distant communication between patients and their practitioners will be shortened. The Saudi FH Database will be coordinated centrally in a manner consistent with accesses to cascade screening in order to ensure authenticity and accuracy of collected data. The minimum common data gathered for the Saudi FH Database will be guided by clinical and laboratory experts. Connection with other databases or registries and clinical cohort, both within Gulf region and worldwide will also ensure harmonisation of the data items.

## 9 GENETIC TESTING AND CASCADE SCREENING

Genetic testing is critical for building the FH registry, as it increases the sensitivity of FH screening and identifies the underlying genotype of the population. Confirmation of FH diagnosis by genetic testing should be made after a referral from a lipid specialist or any equivalent. Screening for genetic mutations will include known FH genes (candidate genes) described above. If no mutations have been identified in these genes, a copy number variants search and whole-exome sequencing would be the next line of action. Cascade is the terminology describing the procedure where FH is explored in relatives of an FH case starting with the patient himself as an index case (the first individual with the clinical presentation of a disease in a pedigree) and then “cascading” throughout the rest of the extended family [55]. In cascade screening, first-, second-, and third-degree relatives, whether affected or not affected, should go through the test as they have 50%, 25% and 12.5% possibility of inheriting the mutation, respectively. Although HoFH can result from consanguineous breeding because of autozygosity, it can also arise from breeding between nonrelated HeFH individuals. Developing a database for such a high prevalent disease can highly improve the early detection of the disease. Furthermore, this project will support and justify universal screening of FH in children in Saudi Arabia (age range 2-9 years) as implemented in other countries.

## CONCLUSION

The successful initiation of the FH registry relies on public awareness, starting with the families, physicians and government officials. Attention to the premature CVD risk associated with FH will eventually push for cascade screening. These objectives can be achieved by a strong pre-registry campaign (*e.g.* via the media) that could also eliminate cultural barriers hindering the establishment of a registry. This will also set the stage for the Gulf Countries Council (GCC) National FH registry and the regional Middle East and North Africa (MENA) FH registry in the long-term.

## Figures and Tables

**Fig. (1):**
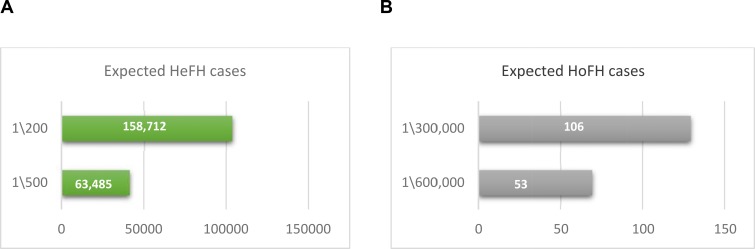
Estimated prevalence of Familial Hypercholesterolemia in Saudi Arabia. **A.** Expected Heterozygous Familial Hypercholesterolemia (HeFH) cases. **B.** Expected Homozygous Familial Hypercholesterolemia (HoFH) cases.

**Fig. (2):**
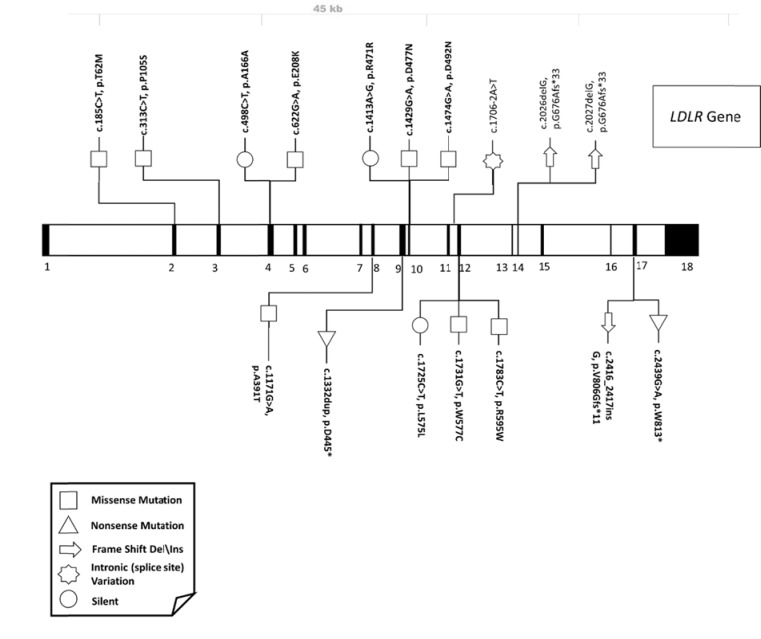
Graphical representation of FH-related variations in the LDLR gene reported from the Saudi population.
